# Aberrantly activated Gli2-KIF20A axis is crucial for growth of hepatocellular carcinoma and predicts poor prognosis

**DOI:** 10.18632/oncotarget.8441

**Published:** 2016-03-28

**Authors:** Chao Shi, Dengliang Huang, Nonghua Lu, Dan Chen, Minhong Zhang, Yehong Yan, Libin Deng, Quqin Lu, Hua Lu, Shiwen Luo

**Affiliations:** ^1^ Center for Experimental Medicine, The First Affiliated Hospital of Nanchang University, Nanchang, Jiangxi, China; ^2^ Department of Gastroenterology, The First Affiliated Hospital of Nanchang University, Nanchang, Jiangxi, China; ^3^ Department of General Surgery, The First Affiliated Hospital of Nanchang University, Nanchang, Jiangxi, China; ^4^ Institute of Translational Medicine, Nanchang University, Nanchang, Jiangxi, China; ^5^ Department of Epidemiology and Biostatistics, School of Public Health, Nanchang University, Nanchang, Jiangxi, China; ^6^ Department of Biochemistry and Molecular Biology and Tulane Cancer Center, Tulane University School of Medicine, New Orleans, LA, USA

**Keywords:** glioma-associated oncogene 2, Forkhead box M1, kinesin family member 20A, transcriptional regulation, hepatocellular carcinoma

## Abstract

Glioma-associated oncogene 2 (Gli2), a primary transcriptional regulator of Hedgehog (Hh) signaling, is essential for hepatocellular carcinoma (HCC) growth and survival. However, the underlying molecular mechanism and crucial downstream targets of Gli2 in human HCC are not fully understood. Here, we report the identification of kinesin family member 20A (KIF20A) as a novel downstream target of Gli2, which is important for HCC proliferation and tumor growth. Inhibition of Hh signaling leads to a remarkable decrease of KIF20A expression in HCC cells, whereas overexpression of Gli2 elevates KIF20A expression by activating Forkhead Box M1 (FoxM1)-MMB complex-mediated transcription of this kinesin gene. Gli2-induced HCC cell growth requires enhanced expression of KIF20A, and knockdown of Gli2 or KIF20A represses the proliferation of HCC cells *in vitro* and *in vivo*. Correlated with these results, analyses of clinical HCC samples show that Gli2, FoxM1 and KIF20A are highly elevated in primary HCC samples and represent significant risk factors for HCC recurrence and survival. Conclusion: KIF20A is an important downstream target gene of Hh signaling. And, the Gli2-KIF20A axis is essential for the proliferation and growth of human HCC cells. Our study also suggests Gli2-KIF20A axis as a potential target for future therapeutic intervention and as an independent prognostic biomarker for HCC.

## INTRODUCTION

Hepatocellular carcinoma (HCC) is the sixth most common primary malignancy worldwide. Although significant advances have been made in diagnosis and treatment, the prognosis of HCC remains poor. High recurrence rate and resistance to radiochemotherapy account for the poor prognosis of HCC [1]. Therefore, a better understanding of molecular insights into the malignancy of HCC is necessary for the development of an effective target-specific therapy against HCC with minimum side effects.

One of the important molecular pathways associated with HCC is Hedgehog (Hh) signaling [2]. Activation of Hh signaling relies on the binding of an Hh ligand to Patched, which unleashes the transmembrane G-protein-coupled receptor Smoothened to activate a family of transcription factors, Glis, by inducing their nuclear translocation. In the nucleus, Glis, specifically Gli2 or Gli3, bind to the consensus sequence 5′-GACCACCCA-3′ to transactivate expression of their target genes, including CCND1, CCND2, BCL2, SNAL1, and Hh self-regulating genes, such as PTCH1 and GLI1 [3]. There are three members in the Gli family, Gli1, Gli2, and Gli3. Gli1 and Gli2 primarily act as transcriptional activators, whereas Gli3 acts as a transcriptional repressor in the Hh signaling pathway [4]. Gene targeting studies in mice have demonstrated that Gli2 is the primary mediators of Hh signaling and is essential for embryogenesis, whereas Gli1 is dispensable for animal development [5]. In a normal adult liver, Hh pathway activity is generally blocked, whereas constitutive activation of this pathway has been described in HCC [2]. Hh signaling has been shown to contribute to the proliferation, invasion, metastasis and autophagy of HCC cells [6, 7], and blockade of Gli2 is sufficient to repress HCC cell growth [8]. Recent studies have shown that Gli2 is involved in the direct regulation of key cell cycle regulators in the G1 phase, including CCND1 and CCND2 [9]. However, it remains unknown if Gli2 regulates the cell cycle by controlling expression of other cell cycle-regulated genes.

Accelerated cell cycles promote cancer cell growth and proliferation, which involve other molecules, such as kinesins, that are important for mitosis. Kinesin superfamily proteins (KIFs), initially identified in 1985 [10], are present in all eukaryotes. Emerging evidence has shown that KIFs play important roles in cell mitosis and meiosis [11]. Sixteen mitotic kinesins are crucial for the development and progression of various cancers [12]. Knockdown of these genes dramatically inhibited tumor cell growth and caused mitotic arrest and apoptosis. However, it remains unclear how the expression of these mitotic KIFs is regulated and which kinesin(s) play(s) a role in the development and progression of HCC.

To address these outstanding questions, we performed a set of bioinformatic analyses and experiments to identify potential target genes of aberrant Hh signaling that are critical for the development of HCC. We identified kinesin family member 20A (KIF20A) as a downstream target gene of Gli2 via FoxM1-MMB complex. Our results demonstrate that the Gli2-KIF20A axis plays a crucial role in HCC growth and progression and could serve as an important biomarker for predicting HCC prognosis in the clinic. Our study also suggests that this axis might serve as a potential target for future development of anti-HCC therapy.

## RESULTS

### KIF20A is a downstream target of Hh signaling

**Figure 1:**
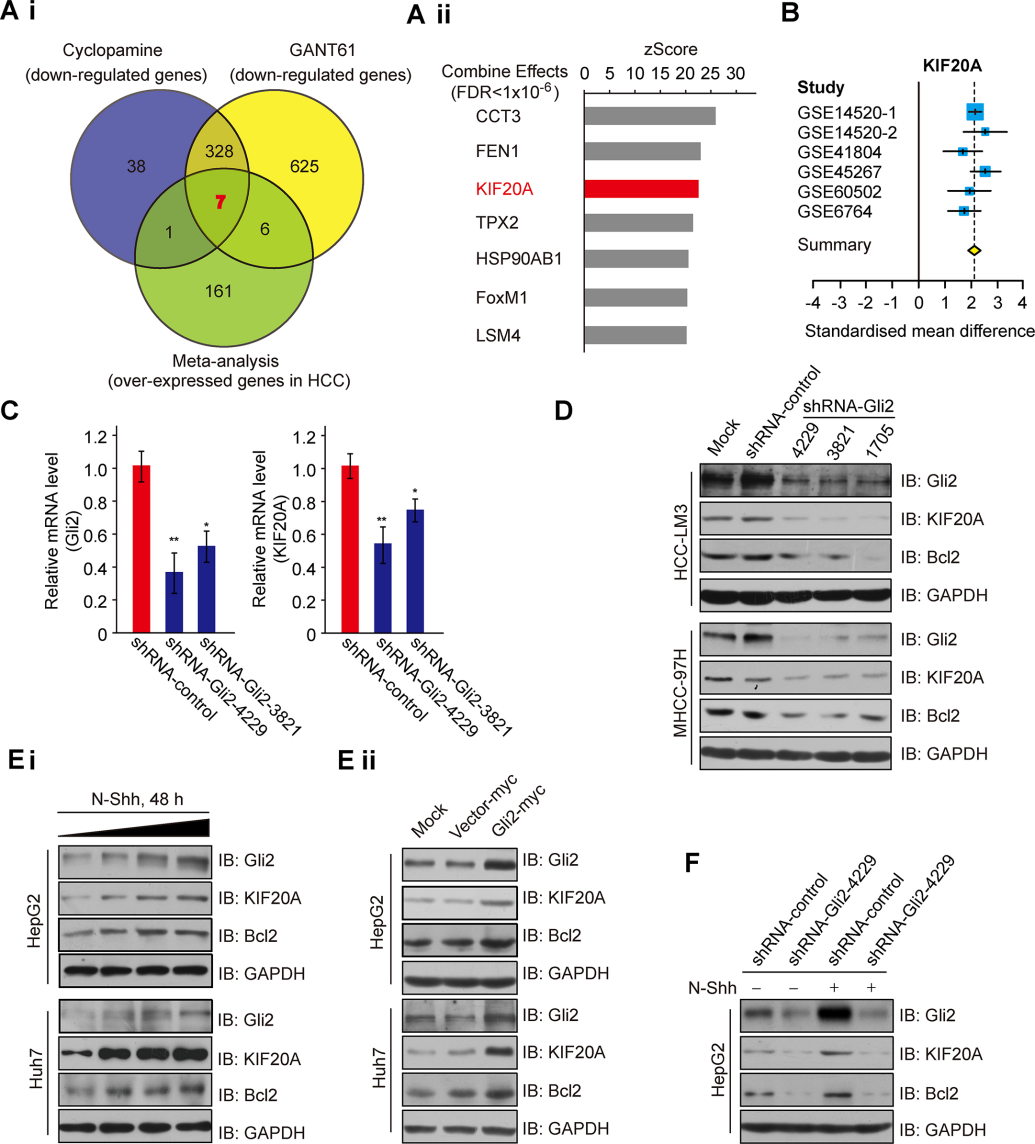
Expression of KIF20A is regulated by the Hh signaling pathway (**Ai**) Venn diagram of Hh inhibitors down-regulated genes in HCC-LM3 cells and overexpressed genes identified by meta-analysis in the HCC cohort. (**Aii**) Seven candidate genes from (Ai) were sorted by z-scores that were extracted from the meta-analysis (Combine Effects). (**B**) Forest plot of KIF20A expression across all meta-analysis datasets. (**C**) HCC-LM3 cells transfected with the indicated plasmids were subjected to real-time PCR analysis for Gli2 and KIF20A expression. *P < 0.05, **P < 0.01. (**D**) HCC-LM3 and MHCC-97H cells were transfected with shRNA-control or shRNA-Gli2 for 48 h and were harvested for WB analysis with the indicated antibodies. (**E**) HepG2 and Huh7 cells were incubated with N-Shh (Ei) or transfected with the indicated plasmids (Eii) for 48 h and were harvested for WB analysis with the indicated antibodies. (**F**) HepG2 cells transfected with shRNA-control or shRNA-Gli2 were incubated with or without N-Shh (0.4 μg/ml) for 48 h and were harvested for WB analysis with the indicated antibodies.

The Hh signaling pathway plays an important role in the cell cycle and proliferation [13]. To investigate the role of aberrant Hh signaling in the initiation and progression of HCC, we analyzed gene expression profiles in response to the inhibition of Hh signaling in HCC-LM3 cells using a cDNA microarray technique. As shown in Supplementary Figure S1A–S1C, 335 genes were down-regulated after treatment with both cyclopamine (an antagonist of SMO) [14] and GANT61 (a specific small-molecule inhibitor of Glis) [15]. To test whether these DEGs induced by Hh signaling inhibition were highly expressed in HCC tissues, we identified six HCC datasets that met the inclusion criteria (368 tumors, 341 non-tumors; Supplementary Table S1) and performed microarray meta-analyses, as described in the Supplementary Information. This approach identified 175 genes significantly over-expressed in HCC across all six datasets. A comparison of these candidate genes with a list of 335 genes that were identified as being markedly repressed (*DiffScore* < −50) in HCC-LM3 cells treated with both cyclopamine and GANT61 revealed 7 putative Hh targets that were overexpressed in HCC (Figure 1A and Supplementary Figure S1D). Among these candidates, KIF20A captured our attention because it is a member of the KIF family, which has been shown to play an important role in the cell division and proliferation of cancer cells [16]. Expression of the KIF20A gene was markedly decreased by more than 50% after blocking Hh signaling (Supplementary Figure S1E, left). This result was further validated by real-time PCR analysis (Supplementary Figure S1E, right). Moreover, KIF20A was significantly overexpressed across all HCC datasets (Figure 1B and Supplementary Figure S1F). Because Gli2 is the primary transcription factor of Hh signaling and plays a predominant role over Gli1 and Gli3 in regulating the expression of downstream genes and the proliferation of HCC cells [8], we also tested whether Gli2 knockdown affects the expression of KIF20A. Gli2 knockdown markedly repressed the mRNA levels of KIF20A (Figure 1C). These results indicate that blockade of Hh signaling significantly represses the expression of KIF20A, which is highly expressed in HCC tissues.

Next, we tested whether protein expression of KIF20A is also reduced in response to inactivation of Hh signaling. Consistent with the above results, both shRNAs-Gli2 and Hh inhibitors (cyclopamine/GANT61) decreased the protein levels of Gli2 and KIF20A, as well as Bcl2 (as a positive control) [17], in HCC-LM3 and MHCC-97H cells (Figure 1D and Supplementary Figure S1G). In line with this result, treatment of HepG2 and Huh7 cells with N-Shh, an Hh ligand, increased the protein levels of Gli2, KIF20A and Bcl2 in these cells (Figure 1Ei), whereas overexpression of Gli2-myc induced higher protein levels of KIF20A and Bcl2 (Figure 1Eii). The induction of KIF20A was dependent on Gli2, as knockdown of the latter markedly blocked the N-Shh induction of the former in HepG2 cells (Figure 1F). Taken together, these results demonstrate that Gli2 is responsible for the induction of KIF20A in response to Hh signaling in HCC cells.

### Gli2 enhances KIF20A expression via activation of FoxM1

**Figure 2:**
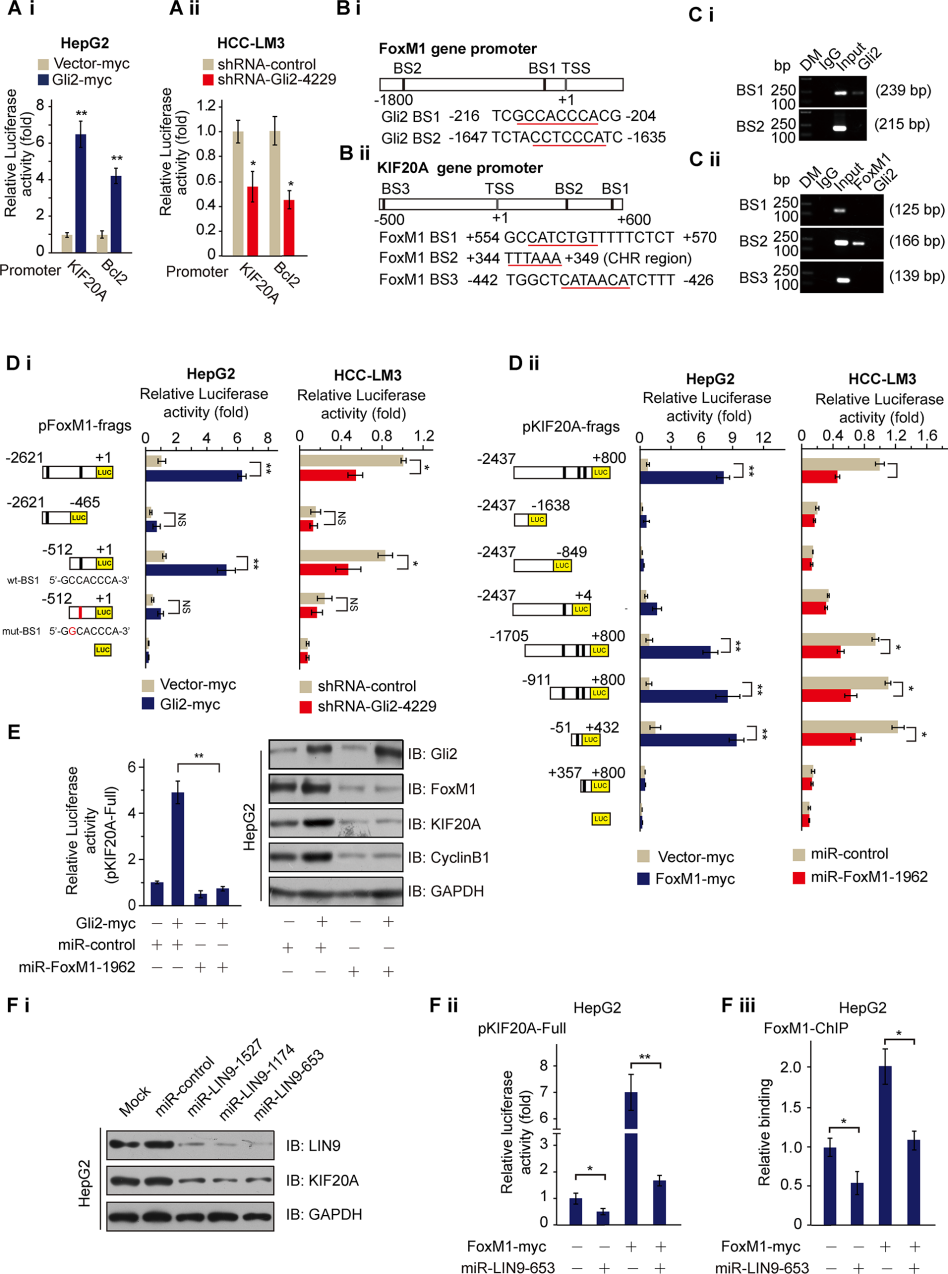
Gli2 regulates the expression of KIF20A by directly promoting FoxM1 expression (**Ai** and **Aii**) KIF20A-luciferase constructs were transfected into HepG2 or HCC-LM3 cells with or without the indicated plasmids for 48 h and subjected to dual luciferase assay. Human Bcl2-luciferase constructs served as the positive control. *P < 0.05, **P < 0.01. (**Bi** and **Bii**) Schematic diagrams of the FoxM1 and KIF20A promoter regions indicate the putative transcription factor binding sites. (**C**) Chromatin was isolated from HCC-LM3 cells, and ChIP assays were performed with control (IgG), anti-Gli2 or anti-FoxM1 antibodies. Specific primers for each putative binding element were used for the PCR analyses. DM, DNA marker. (**Di**) A series of FoxM1-luciferase constructs (left) were transfected into HepG2 or HCC-LM3 cells with or without the indicated plasmids for 48 h and subjected to dual luciferase assay. (**Dii**) A series of KIF20A-luciferase constructs (left) were transfected into HepG2 or HCC-LM3 cells with or without the indicated plasmids for 48 h and subjected to dual luciferase assay. *P < 0.05, **P < 0.01. (**E**) The KIF20A promoter and Gli2-myc or miR-FoxM1-1962 were transfected into HepG2 cells in triplicate. Relative KIF20A promoter activities were measured 48 h after transfection by the dual luciferase assay (left). And the proteins harvested from the cells were subjected to WB analysis (right). **P < 0.01. (**Fi**) HepG2 cells were transfected with miR-control or miR-LIN9 for 48 h and harvested for WB analysis with the indicated antibodies. (**Fii**) The KIF20A promoter and FoxM1-myc or miR-LIN9 were transfected into HepG2 cells in triplicate. Relative KIF20A promoter activities were measured 48 h after transfection by the dual luciferase assay. *P < 0.05, **P < 0.01. (**Fiii**) FOXM1 ChIP was performed on promoters of the KIF20A after knockdown of LIN9 or in the presence of a scramble miR-control. *P < 0.05.

Next, we determined whether Gli2 could directly promote the transcription of KIF20A. Overexpression of ectopic Gli2 markedly increased luciferase activity driven by both the KIF20A and Bcl2 promoters in HepG2 cells (Figure 2Ai), whereas Gli2 knockdown drastically suppressed luciferase activity in HCC-LM3 cells (Figure 2Aii). However, we could not find any putative Gli2-binding DNA elements in the KIF20A promoter using the promoter-identifying program MatInspector professional version 7.2 from Genomatics (http://www.genomatix.de/) [18], suggesting that Gli2 regulates KIF20A transcription through an indirect mechanism.

Previous studies showed that expression of KIF20A is regulated in a cell cycle-dependent manner, as it peaked in the G2/M phase [19]. Also, FoxM1, a member of the Forkhead box transcription factor family, plays a crucial role in the regulation of periodic gene transcription at the G2-M phase of the cell cycle [20]. Additionally, our microarray and meta-analyses showed that FoxM1 is over-expressed in HCC tissues, and its expression is significantly decreased after treatment with cyclopamine and GANT61 (Figure 1A and Supplementary Figure S1D). Thus, we suspected that Gli2 might induce the transcription of KIF20A via FoxM1. Supporting this idea, two potential Gli2-binding sites (BS1: −216 ~ −204 and BS2: −1647 ~ −1635) were identified in the FoxM1 promoter (Figure 2Bi). We also found two consensus FoxM1 binding sites (BS1: +554 ~ +570 and BS3: −442 ~ −426) in the KIF20A promoter as well as a CHR (cell cycle gene homology region) region (BS2: +344 ~ +349) [21, 22] as a potential FoxM1 binding site downstream of the transcriptional start site of the KIF20A gene (Figure 2Bii). To test this idea, we conducted a set of ChIP assays and found that Gli2 binds to BS1, but not BS2, of the FoxM1 promoter, whereas FoxM1 binds to CHR, but not the other two predictive sites, in the KIF20A promoter (Figure 2C).

To confirm the ChIP results, we constructed a set of luciferase reporter vectors driven by either the Gli2-binding site-containing FoxM1 promoter or the FoxM1-binding site-containing KIF20A promoter (Figure 2D) and performed luciferase reporter assays using HCC cells. As shown in Figure 2D, overexpression of Gli2 increased luciferase activity driven by the full-length or the short BS1-containing FoxM1 promoter, but not the pFoxM1–(−2621 ~ −465) region that lacks Gli2 binding sites or the mutated BS1-containing FoxM1 promoter in HepG2 cells (left graph of Figure 2Di), whereas Gli2 knockdown markedly decreased this luciferase activity in HCC-LM3 cells (right graph of Figure 2Di). Additionally, as expected, overexpression of FoxM1 significantly stimulated luciferase activity driven by the FoxM1-binding DNA element-containing KIF20A promoter and its fragments, but not the other fragments that lack CHR region (left graph of Figure 2Dii), whereas FoxM1 knockdown markedly reduced the luciferase activity driven by these promoter regions (right graph of Figure 2Dii and Supplementary Figure S2Ai). These results indicate that the KIF20A–(−51 ~ +432) DNA fragment containing CHR is sufficient for FoxM1-binding. The role of FoxM1 in the Gli2 activation of KIF20A transcription was further confirmed because FoxM1 knockdown impaired the induction of KIF20A promoter-driven luciferase activity and KIF20A protein levels by the overexpression of Gli2 in HepG2 cells (Figure 2E).

MMB is a complex, which includes MuvB core complex components (LIN9, LIN37, LIN52, LIN54, and RBBP4) and MYBL2. And it plays a role in controlling cell cycle dependent transcription of genes at the G2-M phase [23]. Since the CHR element is important for recruitment of the MMB complex, and FoxM1 controls cell cycle dependent gene expression by interacting with the LIN9 member of the MMB core [23, 24], we wondered if FoxM1 is also recruited to the CHR element of the KIF20A promoter to regulate KIF20A expression via LIN9. We first examined the role of LIN9 in FoxM1-mediated transcriptional activation. LIN9 knockdown decreased the protein levels of KIF20A (Figure 2Fi). And overexpression of FoxM1 resulted in the expected activation of KIF20A promoter-driven luciferase reporter gene. However, the activation was severely curtailed upon LIN9 knockdown (Figure 2Fii). Consistently, LIN9 knockdown also markedly reduced FoxM1 binding to the CHR of the KIF20A promoter, indicating that the MMB complex recruit FoxM1 to the CHR element of the KIF20A promoter (Figure 2Fiii). Taken together, these results demonstrate that Gli2 regulates KIF20A expression by activating FoxM1-MMB-mediated transcription of this kinesin gene.

### The Gli2-KIF20A axis contributes to proliferation and cell cycle progression of HCC cells

**Figure 3:**
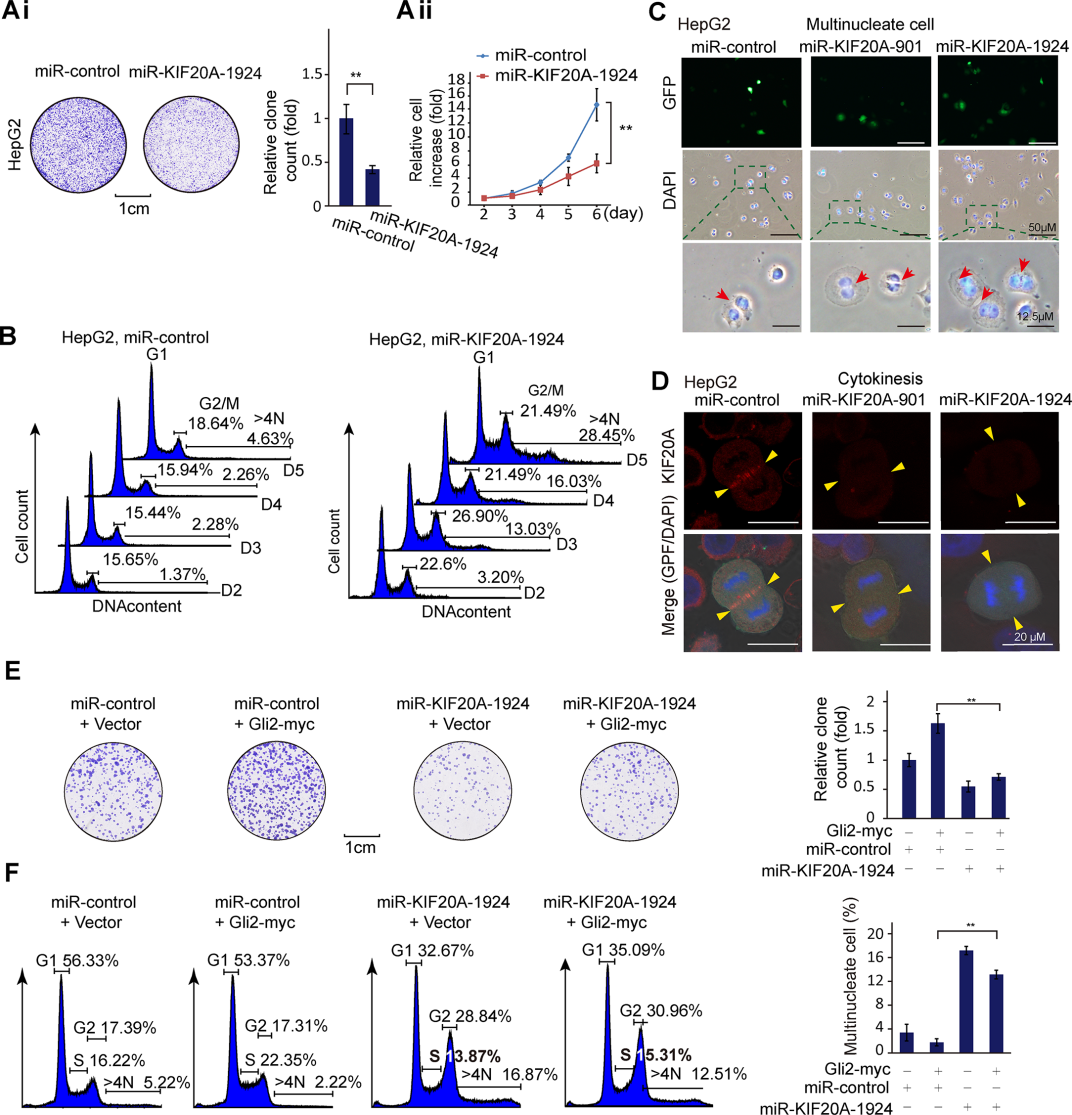
The Gli2-KIF20A axis mediates the growth of HCC cells by promoting cell cycle progression (**Ai**) Representative clonogenic assay of HepG2 cells expressing the indicated plasmids. Quantitative analysis was performed using ImageJ software. The bar graph shows the means ± SD, *n* = 3, **P < 0.01. (**Aii**) HepG2 cells transfected with the indicated plasmids were harvested at the indicated time points, and the numbers of cells were expressed as the fold change relative to the first time point. The data shown represent the means ± SD, *n* = 3, **P < 0.01. (**B**) Cell cycle distributions of HepG2 cells from (Aii) were measured at the indicated time points using flow cytometry. (**C**) HepG2 cells transfected with the indicated plasmids were fixed and stained for DNA (DAPI stain). Binucleated/multinucleated cells were observed (indicated by an arrow). (**D**) HepG2 cells transfected with the indicated plasmids were subjected to an immunofluorescence assay for KIF20A expression. Yellow block arrows indicate the KIF20A location and the regressed cleavage furrows of mitotic cells in KIF20A knockdown cells. (**E**) HepG2 cells transfected with the indicated plasmids were subjected to clonogenic assays. Quantitative analysis was performed using ImageJ software. The bar graph shows the means ± SD, *n* = 3, **P < 0.01. (**F**) HepG2 cells transfected with the indicated plasmids for 72 h were subjected to cell cycle assays. The percentages of multinucleate cells were quantified as a histogram. The data shown represent the means ± SD, *n* = 3, **P < 0.01.

To determine the biological effect of KIF20A on HCC cells, we conducted a set of cell colony and survival assays using two engineered miRNA constructs that efficiently reduced the expression of KIF20A in HepG2 and HCC-LM3 cells (Supplementary Figure S2Aii). As shown in Figure 3A and Supplementary Figure S2B and 2C, KIF20A knockdown reduced the colony formation and growth rate of HepG2 and HCC-LM3 cells. Moreover, knockdown of KIF20A resulted in the accumulation of G2/M, ≥ 4N (≥ 4N cells, i.e., binucleated and multinucleated cells) HepG2 and HCC-LM3 cells (Figure 3B and Supplementary Figure S2D). This phenomenon was confirmed by fluorescence microscopic analysis (Figure 3C). Furthermore, KIF20A knockdown resulted in a later regression of cleavage furrow and subsequently impaired cytokinesis of HepG2 cells, as analyzed by immunofluorescence and time-lapse live-cell imaging (Figure 3D, Supplementary Figure S2E). These results indicate that KIF20A is important for mitosis during the cell cycle of HCC cells.

To determine whether Gli2 promotes the proliferation of HCC cells, we performed colony and flow cytometric assays. As shown in Supplementary Figure S2F, overexpression of Gli2 accelerated cell growth in HCC cells. Consistent with these results, overexpression of Gli2 increased the expression of CyclinD1, CyclinE2 and CyclinB1, as revealed by Western blot analysis (Supplementary Figure S2G). In addition to these cell cycle-regulated proteins, we tested whether KIF20A is also a downstream player in Gli2-promoted cell cycle progression. As shown in Figure 3E and 3F, KIF20A knockdown attenuated the promotion of HCC cell cycle progression by the overexpression of Gli2. These results demonstrate that silencing KIF20A can inhibit HCC cell growth and proliferation by blocking mitosis and also preventing Gli2-promoted cell cycle progression.

### Inhibition of either Gli2 or KIF20A reduces HCC growth *in vivo*

**Figure 4:**
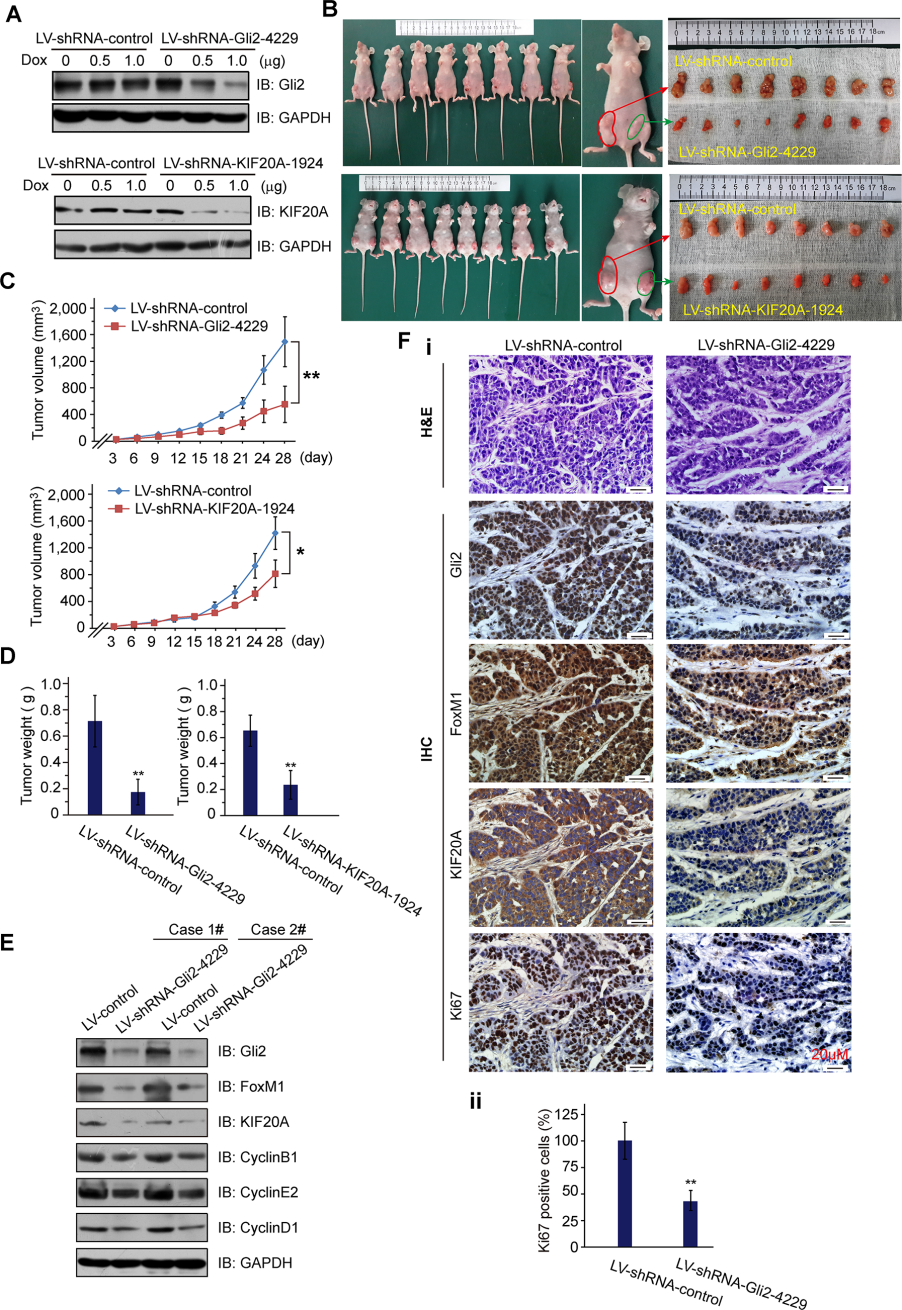
Knockdown of Gli2 or KIF20A suppresses the growth of HCC xenografts (**A**) HCC-LM3 cells were infected by lentivirus with shRNAs (Lenti-shRNA-control and Lenti-shRNA-Gli2 or Lenti-shRNA-KIF20A), and the potency of the Lenti-shRNAs Tet-on system was validated by WB analysis. (B-E) Tumors from the mice described in Supplementary Information were extracted and photographed (**B**). The graphs show the tumor growth (**C, D**). Effective knockdown of Gli2 in the HCC-LM3 cell line was confirmed by WB analysis (**E**), and these cells were examined for FoxM1, KIF20A and markers of the cell cycle (CyclinD1, CyclinE2 and CyclinB1). (**Fi**) Representative H & E, Gli2-, FoxM1-, KIF20A- and Ki67-stained sections from shRNA-control or shRNA-Gli2 expressed xenografts. (**Fii**) Quantification of Ki-67 staining. *n* = 8, **P < 0.01. Scale bars: 20 μm.

To further determine the biological role of the Gli2-KIF20A axis in HCC growth, we established xenograft HCC tumor models by employing three stable doxycycline-inducible HCC-LM3 cell lines that express Lenti-shRNA-control, Lenti-shRNA-Gli2 and Lenti-shRNA-KIF20A (Figure 4A). These animals were split into two groups: group 1, shRNA-control and shRNA-Gli2; group 2, shRNA-control and shRNA-KIF20A. The HCC-LM3 cell lines in each group were injected subcutaneously into either flank of nude mice as shown in Figure 4B. Four weeks after injection of the cells with the induction of shRNA expression by doxycycline, we harvested tumors for further analyses. As shown in Figure 4B–4D, knockdown of either Gli2 or KIF20A significantly reduced tumor volume and weight. Correspondingly, the expression of important cell cycle-regulated proteins, such as CyclinD1, CyclinE2, CyclinB1, and Ki67, was also markedly reduced (Figure 4E–4F). These results demonstrate that the Gli2-KIF20A axis is crucial for the growth and proliferation of HCC cells *in vivo*.

### Gli2, FoxM1 and KIF20A are abnormally elevated in HCC tissues and predict a poor clinical outcome

**Figure 5:**
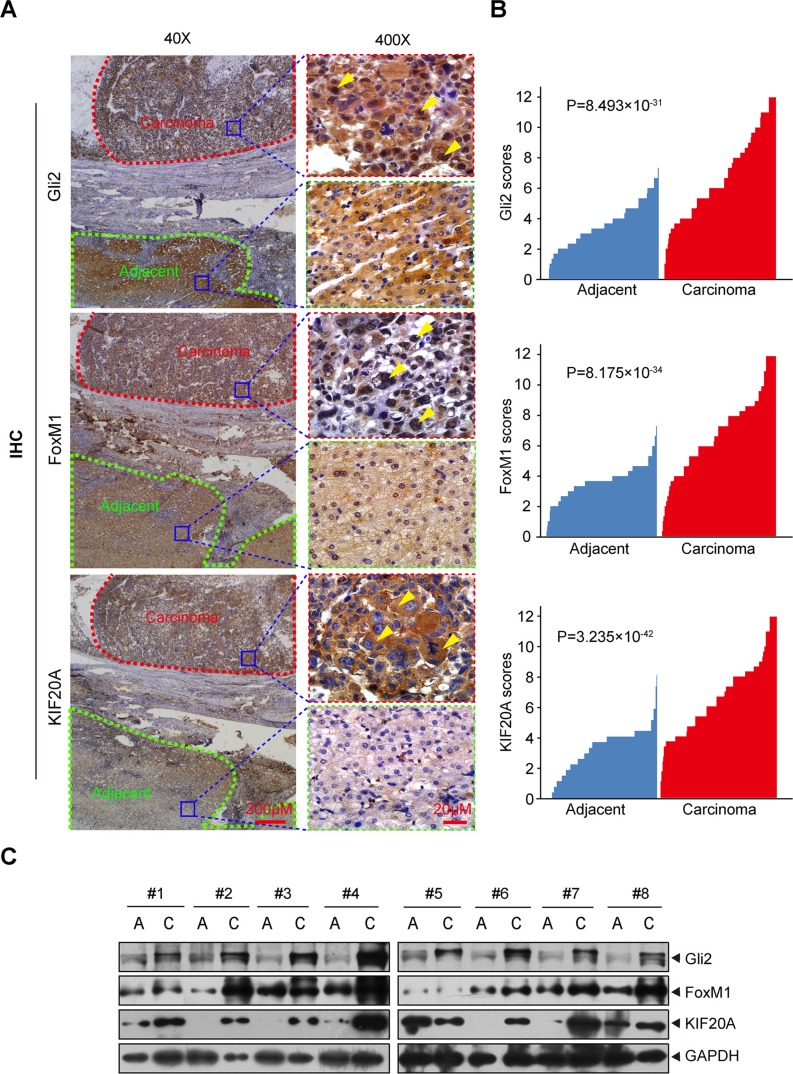
Gli2, FoxM1 and KIF20A are highly expressed in HCC tissues (**A**) IHC detection of Gli2, FoxM1 and KIF20A expression in a representative HCC sample and a matched adjacent normal liver tissue sample. The areas of carcinoma and adjacent tissues were sketched. Subcellular locations of Gli2, FoxM1 and KIF20A were indicated by yellow arrows. (**B**) The expression of Gli2, FoxM1 and KIF20A was plotted using the immunohistochemical scores. Statistical significance was analyzed using the Wilcoxon matched-pairs signed-rank test. (**C**) Eight pairs of randomly selected HCC tissues and adjacent non-tumor tissues were subjected to Western blot analysis for Gli2, KIF20A and FoxM1.

To translate the knowledge gained from the above cell-based and animal studies to the clinic, we examined the protein levels of Gli2, FoxM1 and KIF20A in primary HCC samples and their matched adjacent normal liver tissues (172 pairs plus 38 additional liver cancers without non-tumor tissues) by IHC and Western blot analyses. As shown in Figure 5A–5B, Gli2, FoxM1 and KIF20A were highly expressed in HCC tissues but not in their paired adjacent normal tissues. Eight representative comparisons by Western blot analysis are shown in Figure 5C. These results indicate that Gli2, FoxM1 and KIF20A are highly expressed in primary HCC tissues.

**Figure 6:**
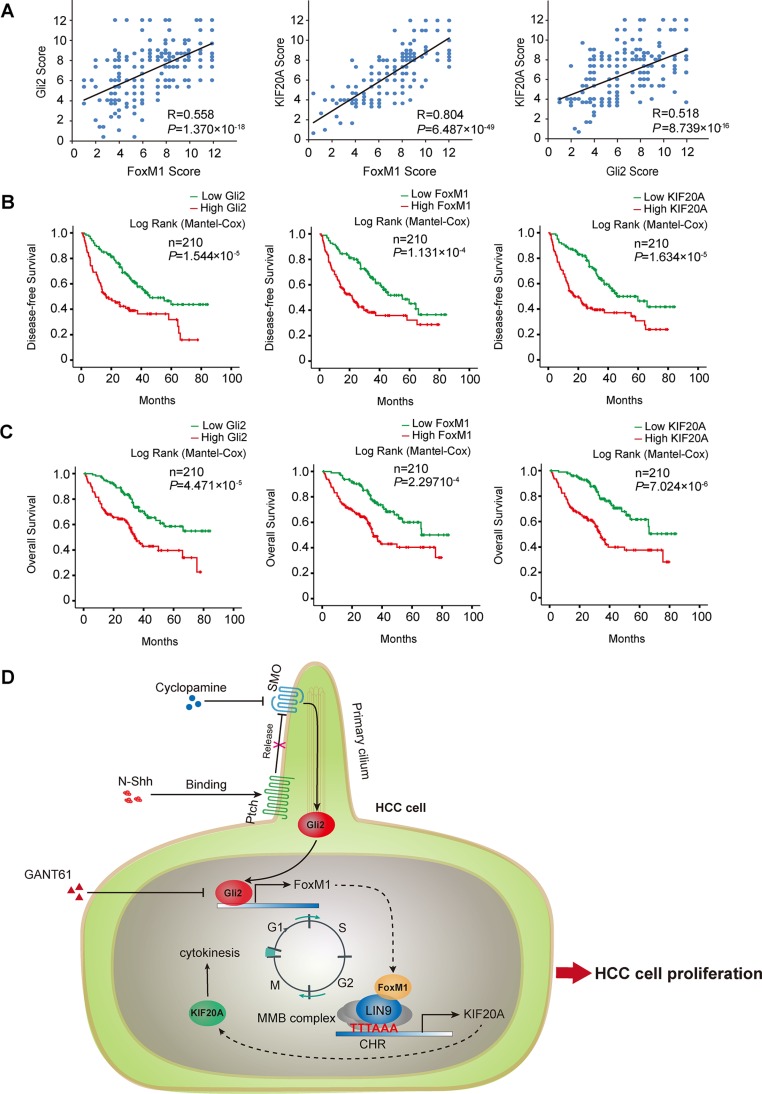
Gli2, FoxM1 and KIF20A are co-expressed in HCC and predict the survival outcome of HCC (**A**) The correlation among Gli2, FoxM1 and KIF20A expression in HCC is displayed as a scatter diagram. The data were analyzed by the Spearman correlation test. (**B, C**) Kaplan-Meier curves were used to represent the difference in the overall survival and disease-free survival of HCC patients between the negative/low and medium/high expression groups for Gli2, FoxM1 and KIF20A. (**D**) A schematic model showing that the Gli2-KIF20A axis regulates the growth of HCC. Hh signaling activates Gli2, which directly transactivates FoxM1. Increased FoxM1 induces KIF20A transcription via the MMB complex, promoting mitosis of the cell cycle and proliferation of HCC cells. The inhibition strategy of the Gli2-KIF20A axis is shown to suppress HCC proliferation and growth in this study.

Next, we analyzed the correlation of the expression of each of the three genes with clinicopathological features in HCC patients. As shown in Table 1 and Supplementary Figure S3, higher expression of Gli2, FoxM1 and KIF20A were all significantly correlated with histologic grade, vascular invasion, HBV infection and TNM stage in HCC. Moreover, the Spearman correlation coefficient showed that expression of Gli2, FoxM1 and KIF20A were positively correlated to each other (Figure 6A). To investigate whether the expression of Gli2, FoxM1 and KIF20A is correlated with the clinical prognosis of HCC, we analyzed two survival parameters, overall survival (OS) and disease-free survival (DFS). The patients were divided into two groups according to the expression levels of Gli2, FoxM1 and KIF20A. The Kaplan–Meier method and log-rank test revealed that HCC patients with higher levels of these three proteins had significantly lower survival rates (Figure 6B, 6C). A univariate analysis also revealed a difference in survival related to histologic grade, vascular invasion, TNM stage, Gli2, FoxM1, and KIF20A status. A multivariate Cox proportional hazards model showed that histologic grade, vascular invasion, Gli2, FoxM1 and KIF20A were significantly associated with tumor recurrence and overall patient survival (Supplementary Table S6). These results demonstrate that Gli2, FoxM1 and KIF20A are co-expressed in HCC tissues and suggest that they can be used as independent prognostic markers for HCC.

**Table 1:** Association of Gli2, FoxM1 and KIF20A expression levels with clinicopathologic characteristics in HCC

Clinicopathologic		Gli2 expression					FOXM1 expression					KIF20A expression				
		Low		High			Low		High			Low		High		
Measurement data	n	Count	%	Count	%	P value	Count	%	Count	%	P value	Count	%	Count	%	P value
**Age, years**																
≤ 50	210	53	25.2%	49	23.3%		47.00	0.22	55.00	0.26		50.00	0.24	52.00	0.25	
>50		60	28.6%	48	22.9%	0.60	50.00	0.24	58.00	0.28	0.97	52.00	0.25	56.00	0.27	0.90
**Sex**																
male	210	95	45.2%	80	38.1%		80	38.1%	95	45.2%		83	39.5%	92	43.8%	
female		18	8.6%	17	8.1%	0.757	17	8.1%	18	8.6%	0.757	19	9.0%	16	7.6%	0.459
**ALB Level, g/L**																
<34	208	9	4.3%	14	6.7%		10	4.8%	13	6.3%		10	4.8%	13	6.3%	
≥34		103	49.5%	82	39.4%	0.133	87	41.8%	98	47.1%	0.748	92	44.2%	93	44.7%	0.572
**AFP Level, μg/L**																
≤20	195	63	32.3%	51	26.2%		58	29.7%	56	28.7%		62	31.8%	52	26.7%	
>20		41	21.0%	40	20.5%	0.522	34	17.4%	47	24.1%	0.220	36	18.5%	45	23.1%	0.171
**CA-199, U/mL**																
≤27	152	63	41.4%	47	30.9%		59	38.8%	51	33.6%		60	39.5%	50	32.9%	
>27		18	11.8%	24	15.8%	0.111	16	10.5%	26	17.1%	0.087	18	11.8%	24	15.8%	0.197
**TBIL, μmol/L**																
≤20.5	210	100	47.6%	79	37.6%		87	41.4%	92	43.8%		87	41.4%	92	43.8%	
>20.5		13	6.2%	18	8.6%	0.151	10	4.8%	21	10.0%	0.092	15	7.1%	16	7.6%	0.982
**Histologic Grade**																
Well or Moderate	210	94	44.8%	65	31.0%		85	40.5%	74	35.2%		90	42.9%	69	32.9%	
Poor		19	9.0%	32	15.2%	**0.006**	12	5.7%	39	18.6%	**1.911×10^−4^**	12	5.7%	39	18.6%	**3.919×10^−5^**
**Vascular invasion**																
Negative	210	109	51.9%	83	39.5%		93	44.3%	99	47.1%		99	47.1%	93	44.3%	
Positive		4	1.9%	14	6.7%	**0.005**	4	1.9%	14	6.7%	**0.033**	3	1.4%	15	7.1%	**0.005**
**HBV infective state**																
Negative	210	27	12.9%	4	1.9%		22	10.5%	9	4.3%		25	11.9%	6	2.9%	
Positive		86	41.0%	93	44.3%	**5.659×10^−5^**	75	35.7%	104	49.5%	**0.003**	77	36.7%	102	48.6%	**1.088×10^−4^**
**Hepatic cirrhosis**																
Negative	209	57	27.3%	45	21.5%		49	23.4%	53	25.4%		51	24.4%	51	24.4%	
Positive		55	26.3%	52	24.9%	0.516	47	22.5%	60	28.7%	0.551	50	23.9%	57	27.3%	0.636
**TNM stage**																
StageI-II	210	89	42.4%	63	30.0%		80	38.1%	72	34.3%		84	40.0%	68	32.4%	
Stage III-IV		24	11.4%	34	16.2%	**0.026**	17	8.1%	41	19.5%	**0.002**	18	8.6%	40	19.0%	**0.002**

## DISCUSSION

Aberrant activation of Hh signaling by Glis is characteristic of a variety of aggressive human carcinomas, including HCC. However, the molecular mechanisms underlying Hh signaling mediating the initiation and progression of HCC cells remain largely elusive. Here, we identified KIF20A as a new signaling axis in the growth of HCC in response to Hh signaling and implicated this pathway as a potential target for future development of anti-HCC therapy.

Microarray technology and meta-analysis have been widely used in the fields of molecular genetics to understand underlying biological mechanisms. And, a combination of the two approaches can increase both the statistical power and generalizability of Microarray analysis [25]. By employing these approaches, we found that FoxM1 and KIF20A were highly expressed in HCC and markedly repressed by blockade of the Hh pathway. Furthermore, we demonstrate that Gli2 regulated KIF20A expression via directly activating FoxM1 expression. Although a previous study found that activation of the Hh pathway is an important determinant of FoxM1 expression in basal cell carcinoma cells [26], our study as presented here for the first time shows that Gli2 directly activates FoxM1 by binding to the Gli consensus sequence (5′-GACCACCCA-3′) in HCC cells. Both Gli1 and Gli2 act as transcriptional activators in the presence of Sonic hedgehog (Shh) ligand, and their DNA binding motifs are similar [4]. However, loss of Gli2 is embryonically lethal, whereas Gli1 is not essential for animal development [27]. Additionally, Gli1 is a direct downstream target of Gli2 and mediates positive feedback regulation in the Hh pathway [28, 29]. Because knockdown Gli1 did not inhibit the growth of HCC cells and had a limited effect on the expression of Hh downstream target genes [8], Gli1 is less likely to act as a primary upstream regulator of FoxM1 in HCC cells. Previous studies have indicated that aberrant activation of Hh signaling by Gli transcription factors plays a critical role in cell proliferation by directly regulating the expression of N-myc, CCND1 and CCND2 genes to promote cell-cycle progression at the G1/S-phase [9, 30]. The Hh signaling has also been proposed to affect CCNE expression by promoting cell-cycle progression through the S-phase [31]. Here, our study revealed that Gli2 communicates with the cell-cycle machinery at the G2/M-phase of HCC cells through the FoxM1-KIF20A axis. Consistently, KIF20A levels are oscillated during the cell cycle, similar to that of cyclin B2, as KIF20A accumulates in mitotic cells, but is essentially absent in interphase cells [19]. Hence, Hh signaling also drives the cell cycle progression through G2/M phase by activating the Gli2-FoxM1-KIF20A pathway.

Also, our study offers additional insight into the mechanism underlying the activation of KIF20A expression by the Hh-Gli2-FoxM1 pathway. Although our finding is somehow similar to a recent study showing that FoxM1 directly regulates KIF20A expression [32], our results are discrepant from theirs in two aspects. First, we found that FoxM1 acts via the CHR elements (hg38: +344 ~ +349, 5′-TTTAAA-3′), instead of the canonical FKH motifs (hg38: +347 ~ +351, 5′-AAATA-3′) that was shown previously [32]. Also, we found that FoxM1 is recruited to this CHR element by interacting with LIN9 (a core component of the MMB complex), rather than binding to the CHR directly. Our finding is also supported by another study, which reported that KIF20A is regulated by the cell cycle-dependant element (CDE) and cell cycle homology region (CHR) [33], though this report did not reveal any specific transcriptional factor that binds to this DNA element. Our finding is further supported by other recent studies showing the functional interaction between FoxM1 and the MMB complex central to the expression of cell cycle-regulated gene independent of the canonical FKH motif [21, 23, 34]. Hence, in concert with other studies, our study logically links the Hh signaling with the regulation of mitotic genes via the Gli2-FoxM1 axis.

Consistent with previous studies [35–39], we found that Gli2, FoxM1 and KIF20A are highly elevated in primary human HCC samples. However, our analysis of 210 HCC cohort samples also revealed that the expression of these genes is positively correlated with each other. Additionally, we found that the expression of Gli2, FoxM1 and KIF20A is significantly correlated with HBV-caused hepatitis, positive vascular invasion, poor histologic grade and TNM stage (Table 1). The expression levels of Gli2, FoxM1 and KIF20A were significantly different between the HBV-positive and HBV-negative HCC patients. This finding is in line with two previous reports [40, 41]. One study showed that chronic hepatitis B virus (HBV) X protein (HBx) expression is correlated with the upregulation of Hh markers in human liver cancer cell lines, HCC samples and an HBx transgenic HCC mice model [40]. Another study showed that HBx also upregulated FoxM1 expression through the ERK/CREB pathway [41]. Finally, our multivariate survival analysis of the expression of these genes in HCC revealed that Gli2, FoxM1 and KIF20A are significantly independent parameters for the prediction of HCC prognosis (DFS and OS).

In summary, we show that Gli2 can directly activate the transcription of FoxM1 in response to Hh signaling, which in turn induces the transcription of KIF20A via the MMB complex in human HCC, establishing a novel Gli2 -KIF20A axis (Figure 6D). And, inhibition of this Gli2-KIF20A axis can efficiently repress HCC tumor growth *in vitro* and *in vivo*. Our results also suggest that the Gli2-KIF20A axis could serve as a potential target for developing therapeutic intervention and as a potential prognostic biomarkers for human HCCs.

## MATERIALS AND METHODS

### Cell lines and transfection

Source and culture of cell lines are described in Supplementary Information. Transient transfection of cells was performed with lipofectamine 2000 (Invitrogen, Carlsbad, CA) according to the instructions of the manufacturer.

### Microarray analysis

HCC-LM3 cells were incubated with cyclopamin (20 μM), GANT61 (20 μM) or vehicle (DMSO) for 48 h. Gene expression profiles were obtained using the genome-wide HumanHT-12 v4 Expression BeadChip arrays (Illumina, San Diego, CA). The methods are described in Supplementary Information. Genes with a *DiffScore* less than −50 or more than 50 were considered differentially expressed genes (DEGs). All raw data are available at Gene Expression Omnibus (GEO) database (Accession number: GSE73481).

### Meta-analysis of gene expression data

CEL data files of gene expression datasets meeting the inclusion criteria were downloaded from NCBI GEO (Supplementary Table S1). Raw data of the datasets were normalized and analyzed using two different meta-analysis methods: combined *p*-values and combined effect size. The leave-one-out meta-analysis was used to control the influence of a single dataset with large samples on meta-analysis results. Details of inclusion criteria and methods are available in Supplementary Information.

### Western blotting and real-time PCR

Protein extracts were obtained using extraction buffer and analyzed by Western blotting as previously described [42]. Quantitative real-time PCR was performed with an ABI step plus one sequence detection system (Applied Biosystems). The specific primers used for PCR amplification are shown in Supplementary Table S2. Details are available in Supplementary Information.

### Chromatin immunoprecipitation (ChIP) assay

A modified protocol from Upstate Biotechnology was used. The predictive binding sequences and the primers used for *FoxM1* and *KIF20A* promoters are listed in Supplementary Table S4. Additional details are available in Supplementary Information.

### Clonogenic, cell proliferation and cell cycle assays

HCC cells were transfected with indicated plasmids and the proliferation rates from day 2 to day 6 were measured using flow cytometry. For the clonogenic assay, HCC cells (3 × 10^3^ per well) were plated in 6-well plates and cultured for 2 weeks. All plates were stained with 0.5% crystal violet (w/v) and the colony numbers were counted. The cell cycle was analyzed by propidium iodide staining and flow cytometry. Details are available in Supplementary Information.

### Lentivirus infection and xenografts

Lenti-X-shRNA Tet-On systems (pGV307-RFP) of shRNA-Gli2 and shRNA-KIF20A were constructed, packed, and purified by GeneChem (Shanghai, China). The shRNA target sequences are listed in Supplementary Table S3. Lentivirus infection was performed according to the protocol provided by the manufacturer. The methods of *in vivo* experiments are described in Supplementary Information. All animal experiments were approved by the Ethical Committee of the First Affiliated Hospital of Nanchang University.

### Patients and clinical samples

This study included 210 patients who were surgically treated for hepatocellular cancer (HCC) at the First Affiliated Hospital of Nanchang University between January 2007 and December 2013. The inclusion criteria were described in Supplementary Information. The study protocol was approved by the Ethics Committee of the Frist Affiliated Hospital of Nanchang University (Nanchang, China).

See Supplementary Information for Antibodies, Reagents, Constructs, Luciferase assay, Time-lapse live-cell imaging, Immunofluorescence, Immunohistochemistry and Statistical analysis.

## SUPPLEMENTARY MATERIALS FIGURES AND TABLES


