# Supplementary material for: Aberrantly activated Gli2-KIF20A axis is crucial for growth of hepatocellular carcinoma and predicts poor prognosis
Source: Oncotarget. 2016 Mar 28;7(18):26206–19. doi: 10.18632/oncotarget.8441 (PMC5041975; doi:10.18632/oncotarget.8441)
Supplement: Supplementary file 1 [file oncotarget-07-26206-s001.pdf]

Aberrantly activated Gli2-KIF20A axis is crucial for growth of hepatocellular carcinoma and predicts poor prognosis

Supplementary Materials

MATERIALS AND METHODS

Cell lines

HEK-293T and human hepatocellular carcinoma cell lines, including HepG2 and Skhep-1, were purchased from the American Type Culture Collection (Manassas, VA). Huh7, HCC-LM3 and MHCC-97H were obtained from the Cell Bank of the Type Culture Collection of the Chinese Academy of Sciences (Shanghai, China). Skhep-1 was cultured in Minimum Essential Media (Hyclone), and HEK293T, Huh7, HepG2, HCC-LM3, and MHCC-97H were cultured in Dulbecco's Modified Eagle's Media (Hyclone) supplemented with 10% FBS (Gibco) and antibiotics (100 U/mL streptomycin and 100 µg/mL penicillin; Invitrogen).

Antibodies, reagents and constructs

The antibodies used in this study include Gli2 (abcam, ab26056), KIF20A (Santa Cruz, sc-374508) and FoxM1 (Abgent, AT2098a) for IHC and WB; Flag (M2) (Sigma, F3165), c-Myc (sigma, M4439), Bcl-2 (Cell Signaling, 2876s), CyclinD1 (BD Biosciences, #556470), CyclinE2 (abcam, ab40890), CyclinB1 (Cell Signaling, 4135S), LIN9 (abcam, ab130360) and GAPDH (Millipore, mAb374) for WB; KIF20A (Abnova, H00010112-B01) for IF; FoxM1 (Santa Cruz, sc502x) for ChIP; and Ki67 (Santa Cruz, SC1540) for IHC. The following reagents were used in this study: cyclopamine was purchased from BioVision (Milpitas, CA), GANT61 was purchased from Sigma (St. Louis, MO), and Lipofectamine 2000 was purchased from Invitrogen (Cambridge, MA). Doxycycline was obtained from Solarbio (Beijing, China). All other chemicals were analytical grade and were purchased from Sigma-Aldrich (St. Louis, MO).

Expression plasmids of human full-length Gli2 (Cat. RC217291) were purchased from OriGene Technologies (Rockville, MD). The human full-length FoxM1 (NM_001095532.1) construct was subcloned into pcDNA3.1-Myc/His (Invitrogen). The shRNA-Gli2 expression vectors were purchased from GeneChem (Shanghai, China). The miRNAi-LIN9, miRNAi-FoxM1 and miRNAi-KIF20A expression vectors were generated using the BLOCK-iT™ Pol II miRNAi Expression Vector Kit (K4936-00, Invitrogen, Carlsbad, CA) according to the manufacturer's protocol [1, 2]. The target sequences of the above shRNA and miRNAi expression constructs are listed in Supplementary Table S3. Recombinant Shh ligand was prepared as described previously and was used at 0.4 µg/ml, unless otherwise indicated [3–5].

Microarray analysis

Total RNA sorted in TRIzol (Invitrogen), double-stranded cDNA and biotin-labeled cRNA were synthesized from total RNA (300 ng) using an Illumina® TotalPrep RNA Amplification Kit (Ambion Inc., Austin, TX) according to the manufacturer's protocol. Quality testing of the cRNA was performed by NanoDrop spectrophotometer and agarose gel electrophoresis. Each biotinylated cRNA (750 ng) was hybridized to an Illumina Human HT expression BeadChip V4 (Illumina Inc., San Diego, CA). The arrays were washed and subsequently scanned using an Illumina® BeadArray Reader.

Meta-analysis of gene expression data

The selection of datasets was based on the following inclusion criteria: each dataset was specific to human hepatocellular cancer; similar case sizes of cancer and non-cancer tissues in each dataset; all series had supplementary CEL data files available; and all datasets were used an Affymetrix U133 GeneChip to minimize platform variation. The raw gene expression data of datasets meeting the inclusion criteria were downloaded from NCBI GEO. The datasets were classified into non-tumor and tumor samples; hepatocellular adenoma samples were excluded. Only probe sets present on all subtypes of Affymetrix U133 platforms were used to describe each sample. Raw data of the datasets were normalized using the Robust Multi-array Average (RMA) algorithm [6]. If multiple probes mapped to a gene, the probe with the most extreme value was used because it was least likely to occur by chance [7].

For the meta-analysis, the normalized datasets were analyzed using two different meta-analysis methods: combined *p*-values and combined effect size. Combined *p*-values were calculated using the directpvalcombi function from the metaMA package (Meta-analysis for MicroArrays) (version 3.1.2) [8] in R (http://www.r-project.org). The combined effect size was calculated using GeneMeta (MetaAnalysis for High Throughput Experiments) (version 1.40) [9] in R (http://www.r-project.org). The leave-one-out meta-analysis was used

to control the influence of a single dataset with large samples on meta-analysis results. We performed both meta-analyses by excluding one dataset at a time, and a stringent threshold (FDR < 1×10^{-6}) was used for selecting differentially overexpressed genes in HCC.

The selection of significant genes was based on the following criteria: (a) meta-effect size > 0 (i.e., overexpressed genes); (b) a Benjamini-Hochberg corrected *p*-value < 1×10^{-6} and a t-statistic > 10 were used to select differentially overexpressed genes in the combined *p*-value method; (c) an FDR < 1×10^{-6} and z-statistic > 20 were used to select differentially overexpressed genes in the combined effect size method. (d) Genes that were significantly overexpressed were identified using leave-one-out meta-analysis.

Western blotting and real-time PCR

Cells were lysed in extraction buffer (0.5% Lubrol-PX, 50 mM KCl, 2 mM $CaCl_2$, 20% glycerol, 50 mM Tris-HCl, and inhibitors of proteases and phosphatases, pH 7.4) and sonicated for three pluses to obtain total cell extracts. Protein extracts were analyzed by immunoblotting. For real-time PCR, total RNA (1 µg) was subjected to reverse transcription using a reverse transcription reagent kit (Applied Biosystems, Foster, CA). Quantitative real-time PCR was performed with an ABI step plus one sequence detection system (Applied Biosystems) [4]. The specific primers used for PCR amplification are shown in Supplementary Table S2. All experiments were repeated at least three times with consistent results.

Chromatin immunoprecipitation (ChIP) assay

A modified protocol from Upstate Biotechnology was used. Briefly, HCC-LM3 cells were fixed at 37°C for 10 min with 1% formaldehyde for cross-linking. The cross-linked cells were re-suspended in 300 µl of ChIP lysis buffer and mixed at 4°C. Then, sonication was performed at level 2 (Ultrasonic Processor, Sonics) for 30 sec to yield fragments from 100 to 400 bp. Eluted DNA was recovered with QIAquick columns (Qiagen) and was used as a template for PCR amplification. The input control was from the supernatant before precipitation. The predictive binding sequences and the primers used for *FoxM1* and *KIF20A* promoters are listed in Supplementary Table S4. The specificity of primers for the different regions of the *FoxM1* and *KIF20A* genes was examined, and no cross-reactive bands were observed.

Construction of luciferase reporter vectors and luciferase assay

The predicted transcription factor Gli2 binding site in the human FoxM1 5′-upstream region (–1800 – +1) and the predicted transcription factor FoxM1 binding site in the human KIF20A 5′-upstream region (–3000 – +800) were analyzed using Genomatix MatInspector software (http://www.genomatix.de/). To construct the reporter vector for the luciferase assay, the 5′-fragment of the human FoxM1 containing Gli2 binding sites –216, –1647, mut –216 and the 5′-fragment of the human KIF20A containing FoxM1 binding sites –442, +334, and +554 were amplified by genomic PCR and were cloned into the firefly luciferase reporter plasmid pGL4.2 (Promega, Madison, WI) using NheI and HindIII restriction sites. The constructed plasmids were designated according to the respective positions of the fragments. Cloned promoter sequences were verified by DNA sequencing. The primers used for the luciferase reporter vectors are listed in Supplementary Table S5.

For the luciferase reporter assays, cells (HepG2 or HCC-LM3) were seeded into 12-well plates the day before transfection. The pGL4.2-Luc reporter plasmids (0.2 µg/well) and the internal control plasmid pRL-TK (5 ng/well) were transfected. Plasmids for the expression of Gli2, miR-Gli2, FoxM1, miR-FoxM1 or empty vector (0.4 µg/well) were co-transfected as indicated. Twenty-four hours after transfection, reporter gene activity was assayed using the Dual Luciferase Assay System (Promega) according to the manufacturer's protocol. All experiments were performed in triplicate.

Clonogenic, cell proliferation and cell cycle assays

Cells were seeded at a density of 100,000 cells/well in six-well plates and were transfected with shRNA-KIF20A-GFP or other plasmids using Lipofectamine 2000. GFP-positive cells were sorted by flow cytometer 24 h after transfection (MoFlo XDP Cell Sorter, Beckman Coulter). Transfected HepG2 and HCC-LM3 cells were plated in 12-well plates. The number of cells was quantified by flow cytometry for 5 days, and the result was shown as the fold increase compared with the number of cells at day 2. For the clonogenic assay, transfected HepG2 or HCC-LM3 cells (3×10^3 per well) were plated in 6-well plates and cultured for 2 weeks. All plates were stained with 0.5% crystal violet (w/v) and were examined under a microscope. The colony number was determined by Image J software. For cell cycle analysis, cells were harvested and fixed in 1% paraformaldehyde for 45 min and 70% ethanol for 4 h at 4°C. Cells were then suspended in 1 ml PBS containing propidium iodide (50 µg/ml) and RNase (50 µg/ml) for 30 min at 37°C in the dark. The cell cycle was analyzed by a flow cytometer, and the data were analyzed using Kaluza 1.3 software (Beckman Coulter).

Time-lapse live-cell imaging and immunofluorescence

HepG2 and HCC-LM3 cells were plated in 12-well polystyrene plates and transfected with miR-KIF20A. After 24 h of culture, cells were treated with 4 mM

thymidine for 24 h to synchronize cells into the G1/S phase, and cells were then washed with PBS and cultured in DMED with 20% FBS for 8 h. The transfected positive cells were discerned by GFP expression. The GFP signal was excited by a 488 nm Argon laser with a 493–565 nm bandpass filter. The culture plates were placed in a 37°C live cell imaging system (LSM 700, Zeiss), and phase-contrast images were acquired using time-lapse recording. For the immunofluorescence experiments, HepG2 cells transfected with miR-KIF20A were grown on glass converslips, fixed in 4% paraformaldehyde for 30 min, permeabilized in ice-cold 0.1% Triton-X-100 for 10 min, and incubated with primary antibody to KIF20A (1:200, Abnova, H00010112-B01) for 16 h at 4°C. After washing 3 times in PBS, cells were incubated with Alexa-594 goat anti-mouse secondary antibody (1:100; Thermo Scientific) for 4 h at 4°C and mounted with VECTASHIELD Antifade Mounting Medium with DAPI (Vector Laboratories). Slides were imaged and analyzed using a Zeiss LSM 700 laser scanning confocal microscope.

Lentivirus infection and xenografts

For Lentivirus infection, 4×10^5 HCC-LM3 cells were incubated with 1×10^8 IU of virus and 8 mg/ml of polybrene (Sigma-Aldrich, St. Louis, MO) for 12 h. Cells were induced in 1 μg/ml doxycycline (Sangon Biotech, Shanghai, China) for 48 h followed by 5 μg/ml puromycin (Sangon Biotech, Shanghai, China) for 14 days to select stably infected cells.

For *in vivo* experiments, 2×10^7 stably infected HCC-LM3 (Lenti-control and Lenti-shRNA-Gli2 or Lenti-shRNA-KIF20A) cells were resuspended in sterile PBS (200 μl) and injected subcutaneously into both flanks of 5-week-old female BALB/c-nu mice (SLAC Laboratory Animal CO. Ltd, Hunan, China). One week after injection, mice were administered 2 mg/ml doxycycline and 5% sterile sucrose in drinking water. The doxycycline-containing water was replenished every 3 days. Tumor sizes in both flanks of mice were measured using Vernier calipers thrice weekly. Tumor volumes were calculated using the formula $V = (L \times W^2)/2$. After 4 weeks, xenografts were harvested for IHC and Western blot analysis. Eight female nude mice (4–5-weeks-old) were used for each group.

Patients and clinical samples

None of the patients had received chemotherapy or radiotherapy before or after surgery. Specimens, including tumors and adjacent non-malignant tissues, were reviewed to confirm the histopathological diagnosis and histologic classifications according to the WHO criteria. HCC cases were staged according to the American Joint Committee on Cancer staging criteria (AJCC, 7th edition, 2009). All demographic data and detailed information on the clinical pathology were collected and summarized.

Immunohistochemistry

Immunohistochemistry analyses were performed as described previously [10]. FFPE tissues from human HCC samples and HCC-LM3 xenografts were cut into 4-μm-thick sections and mounted onto slides. Tissue sections were deparaffinized in dimethylbenzene, rehydrated in a graded alcohol series, and endogenous peroxidase activity was blocked with 0.3% (vol/vol) hydrogen peroxide. Antigens were retrieved by sub-boiling in citrate buffer (0.01 M, pH = 6.0) for 30 min. Subsequently, the slides were rinsed in phosphate-buffered saline, and the sections were incubated overnight at 4°C with Gli2 (1:100), FoxM1 (1:200), KIF20A (1:50), or Ki67 (1:200) primary antibodies in a humidified chamber. After a PBS rinse, the slides were incubated for 30 min at 37°C with appropriate biotinylated immunoglobulins (Zhongshan Biotechnology, China), and immunoreactivity was visualized using a Polink-2 HRP DAB Detection kit (Zhongshan Biotechnology, China) following the manufacturer's procedure. An FSX100 microscope equipped with a digital camera system (Olympus) was used to obtain the IHC images. The expression level of target genes was scored using the German semiquantitative scoring method [11–13]. Each slide was scored for the intensity of nucleic, cytoplasmic and membrane staining (no staining = 0; weak staining = 1; moderate staining = 2; strong staining = 3) and the percentage-positive cells (0% = 0; 1–24% = 1; 25–49% = 2; 50–74% = 3; 75–100% = 4). The final immunoreactive score was determined using the formula: Total score = intensity score multiplied by the extent score. Consecutive sections were stained by H & E to localize cancer tissues and adjacent non-tumor tissues. All samples were evaluated by three investigators who were blind to the pathological diagnosis. For the statistical analysis, immunoreactive scores were grouped into two categories, with scores of 0 to 6 considered Negative/Low expression and > 6 to 12 as Median/High expression.

Statistical analysis

Differences in quantitative data between two groups were analyzed using 2-sided paired or unpaired Student *t*-tests. The intraclass correlation coefficients of IHC scores between two proteins were analyzed by the Spearman correlation coefficient and were assessed by the following guidelines: a coefficient of reliability > 0.75 indicates 'strong' agreement; between 0.4 and 0.75, 'good' agreement; and < 0.4, 'poor' agreement [14]. Specific comparison of IHC scores between two or three independent groups was performed using the Mann Whitney *U* test or the Kruskal-Wallis *H* test, respectively. The χ^2 test was used to analyze the correlation of gene expression and clinicopathological characteristics. For the survival analysis, the Kaplan–Meier method and the log-rank test were used. The Cox proportional hazards model was used to determine the independent factors influencing survival and recurrence

based on the variables selected from the univariate analysis. $P < 0.05$ was considered to be statistically significant. All of the analyses were performed using SPSS software version 18.0 (SPSS, Chicago, IL, USA).

REFERENCES

1. Luo SW, Zhang C, Zhang B, Kim CH, Qiu YZ, Du QS, Mei L, Xiong WC. Regulation of heterochromatin remodelling and myogenin expression during muscle differentiation by FAK interaction with MBD2. EMBO J. 2009; 28:2568–2582.
2. Luo S, Zhang B, Dong XP, Tao Y, Ting A, Zhou Z, Meixiong J, Luo J, Chiu FC, Xiong WC, Mei L. HSP90 beta regulates rapsyn turnover and subsequent AChR cluster formation and maintenance. Neuron. 2008; 60:97–110.
3. Yan R, Peng X, Yuan X, Huang D, Chen J, Lu Q, Lv N, Luo S. Suppression of growth and migration by blocking the Hedgehog signaling pathway in gastric cancer cells. Cell Oncol (Dordr). 2013; 36:421–435.
4. Chen Q, Xu R, Zeng C, Lu Q, Huang D, Shi C, Zhang W, Deng L, Yan R, Rao H, Gao G, Luo S. Down-regulation of Gli transcription factor leads to the inhibition of migration and invasion of ovarian cancer cells via integrin beta4-mediated FAK signaling. PLoS One. 2014; 9:e88386.
5. Tang X, Deng L, Chen Q, Wang Y, Xu R, Shi C, Shao J, Hu G, Gao M, Rao H, Luo S, Lu Q. Inhibition of Hedgehog signaling pathway impedes cancer cell proliferation by promotion of autophagy. Eur J Cell Biol. 2015; 94:223–233.
6. Irizarry RA, Hobbs B, Collin F, Beazer-Barclay YD, Antonellis KJ, Scherf U, Speed TP. Exploration, normalization, and summaries of high density oligonucleotide array probe level data. Biostatistics. 2003; 4:249–264.
7. Ramasamy A, Mondry A, Holmes CC, Altman DG. Key issues in conducting a meta-analysis of gene expression microarray datasets. PLoS Med. 2008; 5:e184.
8. Marot G, Foulley JL, Mayer CD, Jaffrezic F. Moderated effect size and P-value combinations for microarray meta-analyses. Bioinformatics. 2009; 25:2692–2699.
9. Choi JK, Yu U, Kim S, Yoo OJ. Combining multiple microarray studies and modeling interstudy variation. Bioinformatics. 2003; 19:i84–90.
10. Tao Y, Shen C, Luo S, Traore W, Marchetto S, Santoni MJ, Xu L, Wu B, Shi C, Mei J, Bates R, Liu X, Zhao K, et al. Role of Erbin in ErbB2-dependent breast tumor growth. Proc Natl Acad Sci U S A. 2014; 111:E4429–4438.
11. Remmele W, Schicketanz KH. Immunohistochemical determination of estrogen and progesterone receptor content in human breast cancer. Computer-assisted image analysis (QIC score) vs. subjective grading (IRS). Pathol Res Pract. 1993; 189:862–866.
12. Pan X, Zhou T, Tai YH, Wang C, Zhao J, Cao Y, Chen Y, Zhang PJ, Yu M, Zhen C, Mu R, Bai ZF, Li HY, et al. Elevated expression of CUEDC2 protein confers endocrine resistance in breast cancer. Nat Med. 2011; 17:708–714.
13. Zaineddin AK, Vrieling A, Buck K, Becker S, Linseisen J, Flesch-Janys D, Kaaks R, Chang-Claude J. Serum enterolactone and postmenopausal breast cancer risk by estrogen, progesterone and herceptin 2 receptor status. Int J Cancer. 2012; 130:1401–1410.
14. Coenraads PJ, Van Der Walle H, Thestrup-Pedersen K, Ruzicka T, Dreno B, De La Loge C, Viala M, Querner S, Brown T, Zultak M. Construction and validation of a photographic guide for assessing severity of chronic hand dermatitis. Br J Dermatol. 2005; 152:296–301.

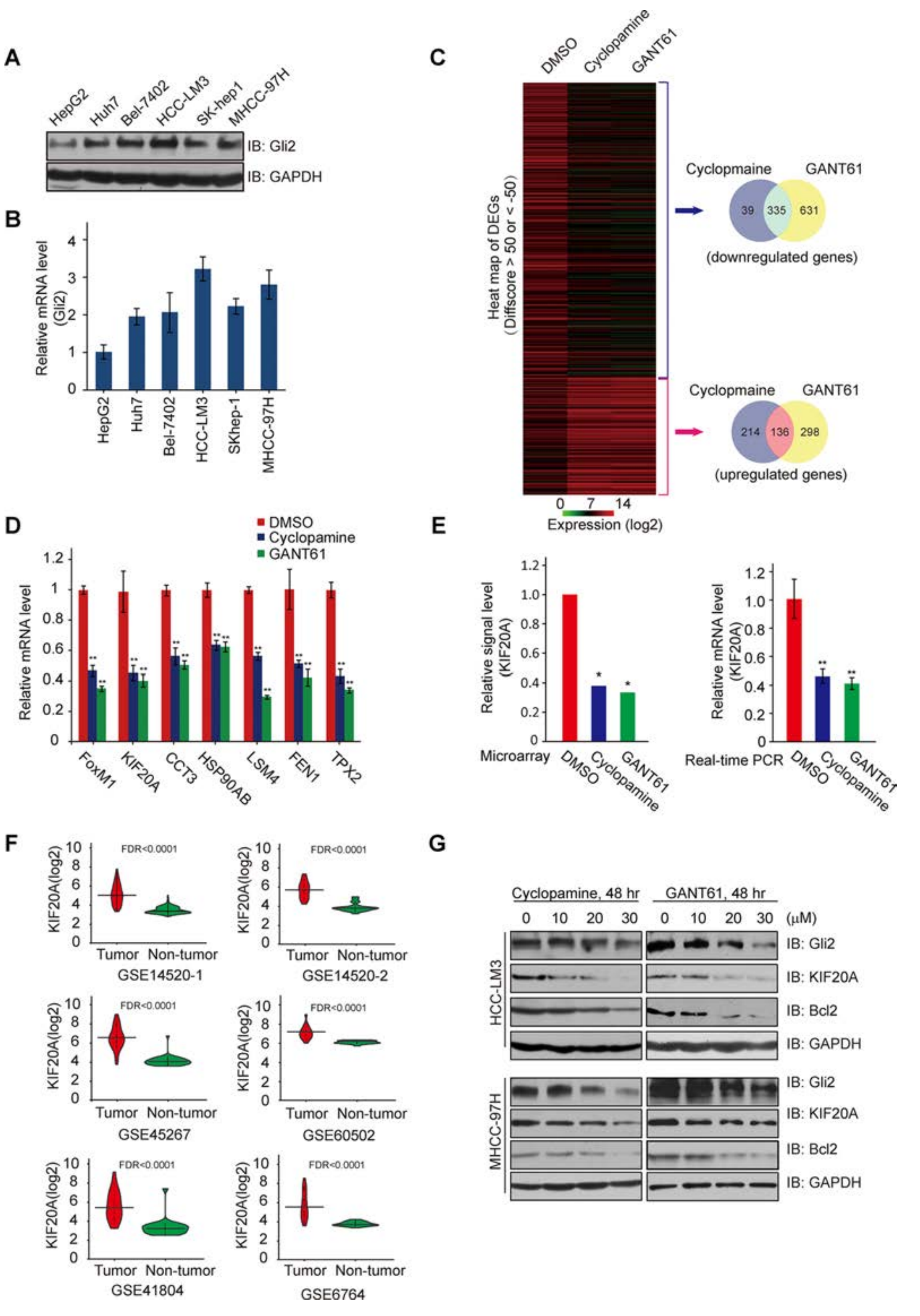

Supplementary Figure S1: Expression of KIF20A genes is markedly suppressed by blockade of Hh signaling. (**A, B**) WB analysis of Gli2 protein and real-time PCR analyses of Gli2 mRNA in human HCC cell lines. The data represent the means ± SD. $n = 3$. (**C**) HCC-LM3 cells were treated with DMSO, cyclopamine or GANT61 for 48 h, and total RNA was extracted. Changes in gene expression were determined by cDNA microarray gene profiling using the Illumina Human HT expression BeadChip V4 (Illumina Inc., San Diego, CA). The common, differentially expressed genes (DEGs) (*DiffScore* ≥ 50 or ≤ –50) corresponding to treatment with cyclopamine and GANT61 compared with DMSO treatment are shown in a heat map (left) and Venn diagrams (right). (**D**) HCC-LM3 cells were treated with either cyclopamine or GANT61 for 48 h and harvested for real-time PCR analysis with the indicated primers, $^{**}P < 0.01$. (**E**) HCC-LM3 cells were treated with either cyclopamine or GANT61 (20 μM, 48 h). The expression of KIF20A was quantified by real-time PCR. The data represent the means ± SD of three determinations, and GAPDH was used to normalize the relative mRNA levels. (**F**) Violin plots were used to show the distribution of KIF20A expression in HCC tissues and non-tumor tissues in each dataset. Line, median; Spot, expression of each case. The R package "Significance analysis of microarrays (SAM)" algorithm was used to determine statistical significance. (**G**) HCC-LM3 and MHCC-97H cells were treated with either cyclopamine or GANT61 (10~30 μM) for 48 h and were harvested for Western blot (WB) analysis with the indicated antibodies.

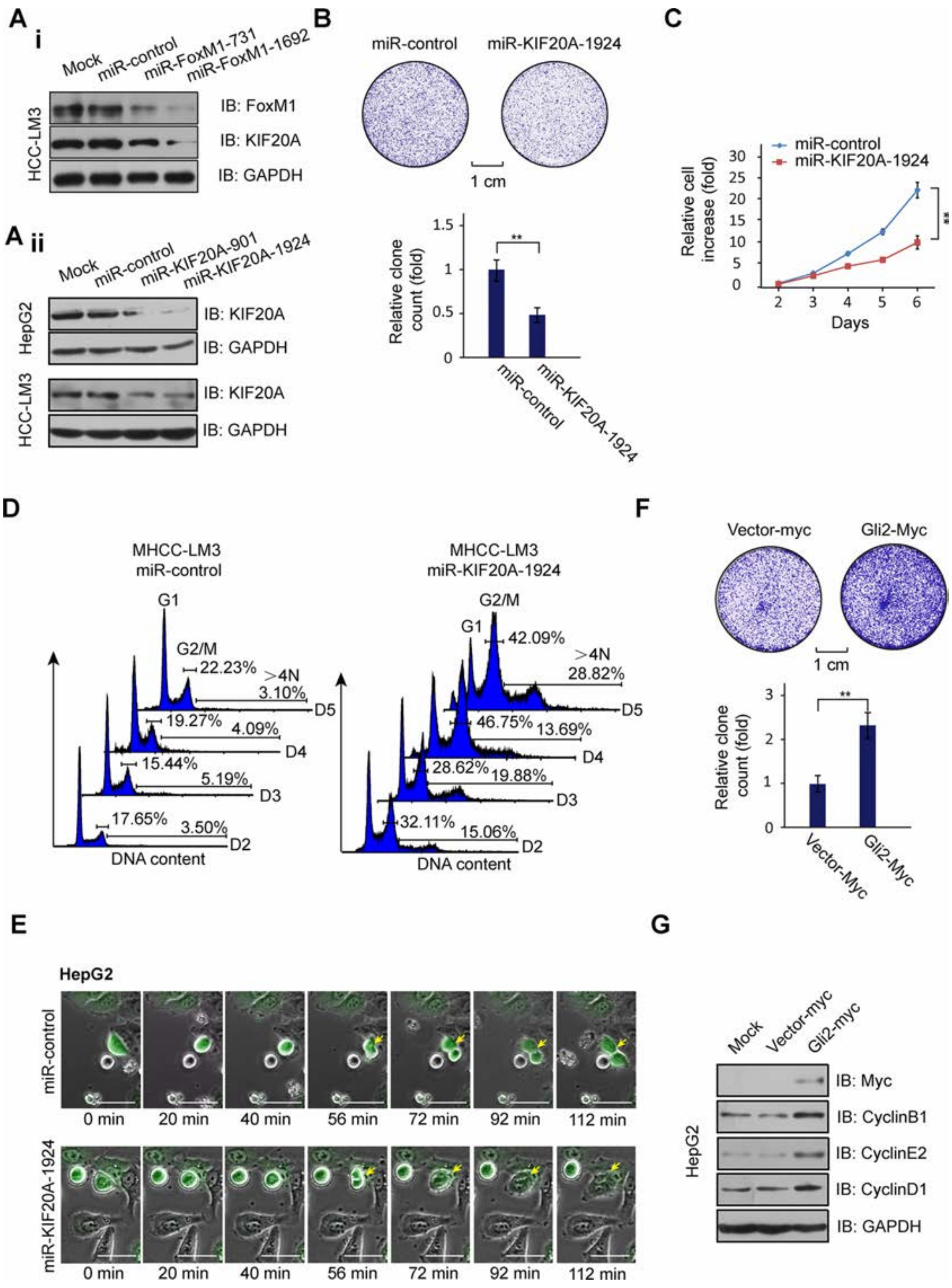

Supplementary Figure S2: KIF20A knockdown results in the failure of cytokinesis and production of binucleated/ multinucleated cells. (**Ai**) HCC-LM3 cells were transfected with the engineered miRNA constructs (miR-control or miR-FoxM1) for 48 h, and the knockdown efficiency of FoxM1 and KIF20A expression were assessed by Western blot analysis. (**Aii**) HepG2 and HCC-LM3 cells were transfected with the engineered miRNA constructs miR-control or miR-KIF20As for 48 h, and the knockdown efficiency of KIF20A was assessed by Western blot analysis. (**B**) Representative clonogenic assay of HCC-LM3 cells expressing the engineered miR-control or miR-KIF20A-1924 constructs. Quantitative analysis was performed using ImageJ software. The bar graph shows the means ± SD, $n = 3$, $^{**}P < 0.01$. (**C**) HCC-LM3 cells transfected with miR-control or miR-KIF20A-1924 were harvested at the indicated time points, and the number of cells was expressed as the fold change relative to the first time point. The data shown represent the means ± SD, $n = 3$, $^{**}P < 0.01$. (**D**) HCC-LM3 cells were transfected with miR-control and miR-KIF20A-1924. Cell cycle distributions were measured at the indicated time points using flow cytometry. (**E**) Time-lapse recording of the failure of cytokinesis observed in HepG2 cells expressing miR-KIF20A-1924. Yellow arrows indicate the regressed cleavage furrows of mitotic cells at the later phase of cytokinesis. (**F**) Representative clonogenic assay of HCC-LM3 cells transfected with empty vector or Gli2-myc plasmids. The cell numbers were expressed as the fold change relative to the first time point. The bar graph shows the means ± SD, $n = 3$, $^{**}P < 0.01$. (**G**) HepG2 cells transfected with empty vector or Gli2-myc plasmids for 48 h were subjected to WB analysis for CyclinD1, CyclinE2, and CyclinB1 expression.

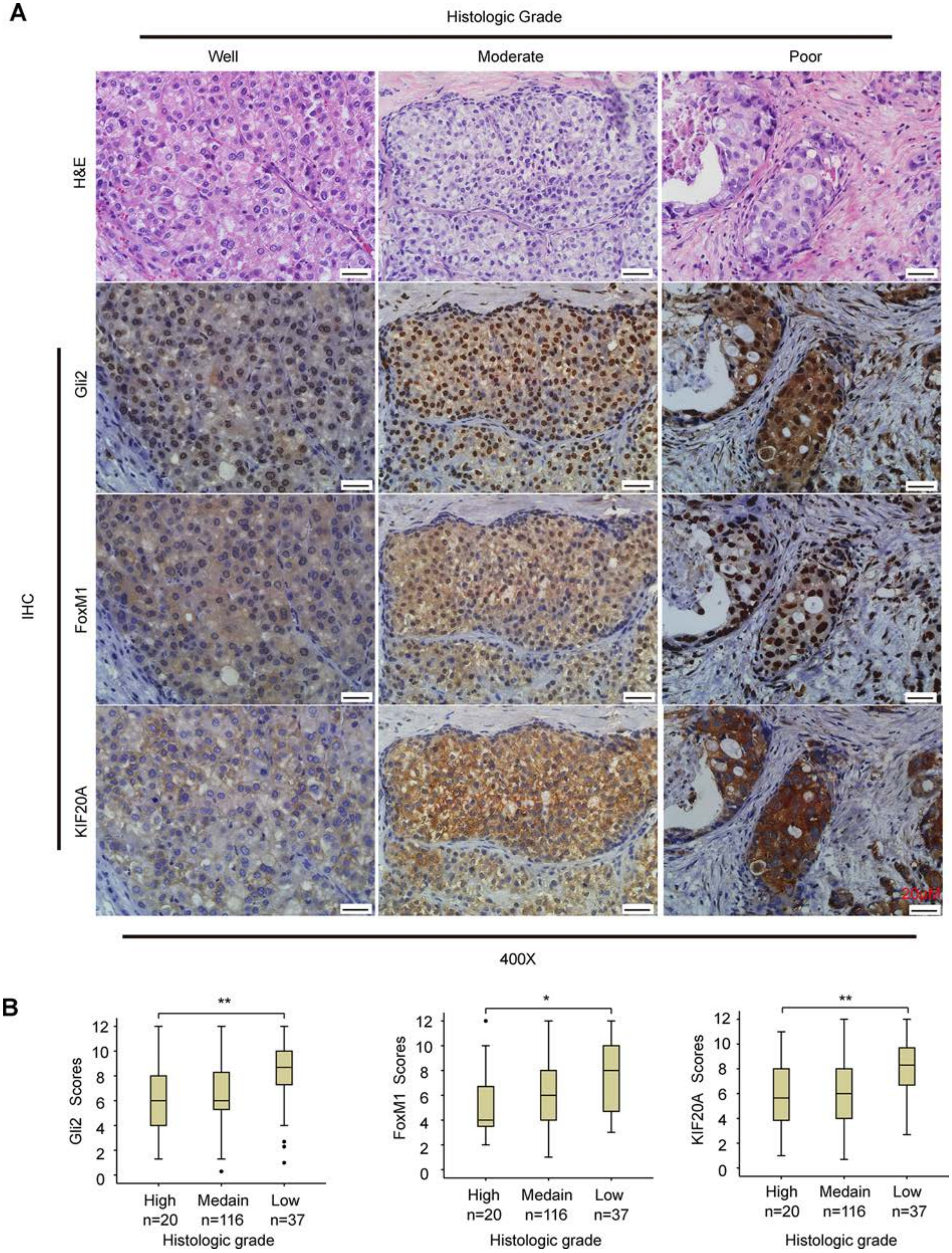

Supplementary Figure S3: Gli2, FoxM1 and KIF20A levels in HCC are closely associated with poor tumor differentiation. (**A**) Correlation of Gli2, FoxM1 and KIF20A expression with the pathological grade of tumors. Three serial sections of HCC tissues were immunohistochemically stained with anti-Gli2, FoxM1 and KIF20A antibodies. Representative images from three cases with different levels of histological differentiation (Well to Poor) are shown. (**B**) Expression scores of Gli2, FoxM1 and KIF20A are shown as box plots, as described in the Supplemental Materials and Methods. The sample numbers of the different grades are shown below the respective group. The data were analyzed by the Kruskal-Wallis H test, *$P < 0.05$, **$P < 0.01$.

Supplementary Table S1: HCC datasets downloaded from NCBI GEO

GEO ID	Platforms	Total	Tumor	Non-tumor
GSE45267	HG-U133_Plus_2	87	48	39
GSE41804	HG-U133_Plus_2	40	20	20
GSE14520–2	HG-U133A_2	43	22	21
GSE14520–1	HT_HG-U133A	445	225	220
GSE60502	HG-U133A	36	18	18
GSE6764	HG-U133_Plus_2	58	35	23
Total		709	368	341

Supplementary Table S2: Primers used for real-time PCR amplification

Genes	Forward primer (5′ to 3′)	Reverse primer (5′ to 3′)
KIF20A	5′-AGCACCCTAAACCAGTTACCC-3′	5′-CTGTCGTGGATTCGCACTTA-3′
Gli2	5′-CTGTGGGTTAGGGATGGACTG-3′	5′-GTAAAGTGGGTGGACGTTGCA-3′
Bcl2	5′-CAAACAAGACGCCAACATTC-3′	5′-CTGGAGGTGAAAGCTAGACA-3′
FoxM1	5′-CTGCTTGCCAGAGTCCTTT-3′	5′-CTCCACCTGAGTTCTCGTCA-3′
CCT3	5′-AATGGTGAGACGGGTACTTTG-3′	5′-TGGCCTGAAACGATGTCATC-3′
FEN1	5′-ACCCCGAACCAAGCTTTAG-3′	5′-CTCTTGATGTCATTCTCCCGG-3′
TPX2	5′-GAAGAGAATGGCTGAGGTAGAAG-3′	5′-CTGGTACTTGCGTATTGGATTTG-3′
HSP90AB1	5′-TTGAGAACCTCTGCAAGCTC-3′	5′-TCATGATCCGCTCCATATTGG-3′
LSM4	5′-ACCATGCTTCCCTTGTCAC-3′	5′-TGTTCATCCAGTTGTCGCAG-3′
GAPDH	5′-GAAGGTGAAGGTCGGAGTC-3′	5′-GAAGATGGTGATGGGATTTC-3′

Supplementary Table S3: The shRNAs or miRNAis used for specific genes knockdown

ID	Target sequences (5′-3′)	Start	Remark
shRNA-Gli2(#1)	5′-TCCTGAACATGATGACCTA-3′	4229	for Lentivirus shRNA Tet-On System
shRNA-Gli2(#2)	5′-TCTACTACTACGGCCAGAT-3′	3821	
shRNA-Gli2(#3)	5′-CTCAAGGATTCCTGCTCAT-3′	1705	
miRNAi-KIF20A(1#)	5′-CTTCTTCAACCTAACTGTGAA-3′	901	
miRNAi-KIF20A(2#)	5′-CCGTTCCTGCATGATTGTCAA-3′	1924	for Lentivirus shRNA Tet-On System
miRNAi-FoxM1(1#)	5′-GTGAATCTTCCTAGACCACCT-3′	731	
miRNAi-FoxM1(2#)	5′-CCCCAACCCGGTGTGTCTCGG-3′	1692	
miRNAi-LIN9(1#)	5′-CCTCTCCAGTCACCAATTATA-3′	653	
miRNAi-LIN9(2#)	5′-AGGAGGAGACCTGAATTCCTT-3′	1174	
miRNAi-LIN9(3#)	5′-CATTGCTGACCAGCATTATAT-3′	1527	

Supplementary Table S4: Primers used for ChIP

Gene	Predictive BS (5′ to 3′)	Forward primer (5′ to 3′)	Reverse primer (5′ to 3′)
	BS1: 5′- TCGCCCACCCACG-3′	5′- TGTCGCCTGGCGTGACCAGC -3′	5′- CGGGCTCGAAGGCTGTGCGG -3′
	BS2: 5′- TCTACCTCCCATC-3′	5′- CTTGGTCAGGGAATAGTG -3′	5′- CTCCAGGCAAGAACTGCT -3′
KIF20A			
	BS1: 5′- GCCATCTGTTTTTCTCT -3′	5′- AATAGGCTTTAAGAAATCGGAGCT -3′	5′- TGTGGAGGTGGTGTCAGGATC -3′
	BS2: 5′- TTTAAA -3′	5′- GTCAAGCAGAAGCGAACGACTG -3′	5′- CAGAGCACAACTCCGCCCAC -3′
	BS3: 5′- TGGCTCATAACATCTTT -3′	5′- AAAATGAATGGCTCTTTGAGG -3′	5′- GAGAAGCAGCTTAGTGAATCCTA -3′

BS: Binding Sequences.

Supplementary Table S5: Primers used for construction of luciferase reporter constructs

Promoters	Range	Forward primer (5′ to 3′)	Reverse primer (5′ to 3′)
pKIF20A-full	−2437 – +800	5′-CTAGCTAGCAACAAAAAAAAGTCCAGAAGAGCACGTATC-3′	5′-CCCAAGCTTGCAAAGGGGAAGGGAATGAGAGAAGT-3′
pKIF20A-frag1	−2437 – −1638	5′-CCGCTCGAGTCTACAGAGACAAGAGAGTTGGCTGGGT-3′	5′-CCCAAGCTTTTTGAGACAAGATTGCACTCTGTGGCC-3′
pKIF20A-frag2	−2437 – −849	5′-CCGCTCGAGTCTACAGAGACAAGAGAGTTGGCTGGGT-3′	5′-CCCAAGCTTAATTCACATGCAACGAATAGGTACCCC-3′
pKIF20A-frag3	−2437 – +4	5′-CCGCTCGAGTCTACAGAGACAAGAGAGTTGGCTGGGT-3′	5′-CCCAAGCTTAAAACGACATTTCCCAGGAGGCACCG-3′
pKIF20A-frag4	−1705 – +800	5′-CCGCTCGAGGTTGCAGTGAGCCAAGATCACACCACTG-3′	5′-CCCAAGCTTGCAAAGGGGAAGGGAATGAGAGAAGT-3′
pKIF20A-frag5	−911 – +800	5′-CCGCTCGAGCCTCTCCAAGTGAAATATCACCAGTGAC-3′	5′-CCCAAGCTTGCAAAGGGGAAGGGAATGAGAGAAGT-3′
pKIF20A-frag6	−51 – +432	5′-CCGCTCGAGCTGGGAAATAGTGTACCGGCGTTGGTT-3′	5′-CCCAAGCTTACACCTAGTCGCCGAAGCTGGACTT-3′
pKIF20A-frag7	+357 – +800	5′-CCGCTCGAGATCTGTAACAAAGCTGCACCTCGTGG-3′	5′-CCCAAGCTTGCAAAGGGGAAGGGAATGAGAGAAGT-3′
pFoxM1-full	−2621 – +1	5′-CGGGGTACCAGTGACAGAGCCAGAACCTTGTTTC-3′	5′-CCGCTCGAGCAGTTTGTTCCGCTGTTTGAAATTGG-3′
pFoxM1-frag1	−2621 – −465	5′-CGGGGTACCGTAGGGTTCATGGTGCCG-3′	5′-CCGCTCGAGAGTTTGTTCCGCTGTTTG-3′
pFoxM1-frag2	−512 – +1	5′-CGGGGTACCCCTGTATTTCAGTATCCACCTA-3′	5′-CCGCTCGAGTCCGCTTTCTTCCATCTT-3′

Supplementary Table S6: Univerate and multivariate analysis of factors associated with survival and recurrence of HCCs recurrence

	Disease free survival							Overall survival						
	Univariate analysis			Multivariate analysis				Univariate analysis			Multivariate analysis			
	HR	95% CI	*P*-value	HR	95% CI	*P*-value		HR	95% CI	*P*-value	HR	95% CI	*P*-value	
Age (≤ 50 versus > 50 years)	1.104	0.758 ~1.607	0.607					1.103	0.717 ~ 1.697	0.655				
Sex (male versus famale)	0.796	0.468 ~ 1.354	0.399					0.671	0.346 ~ 1.299	0.236				
ALB (≥ 34 versus < 34g/L)	0.761	0.441 ~ 1.312	0.326					0.677	0.367 ~ 1.250	0.213				
AFP (≤ 400 versus > 400 ug/L)	1.059	0.707 ~ 1.560	0.782					0.974	0.603 ~ 1.571	0.913				
CA-199 (≤ 27 versus > 27U/mL)	1.530	0.942 ~ 2.487	0.086					1.306	0.728 ~ 2.344	0.370				
TBIL (≤ 20.5 versus > 20.5 umol/L)	1.430	0.871 ~ 2.350	0.158					1.370	0.782 ~ 2.398	0.271				
Histologic Grade (grade1-2 versus grade3)	1.941	1.287 ~ 2.928	0.002	1.552	1.010 ~ 2.386	**0.045**	†	2.782	1.771 ~ 4.369	8.894×10^{-6}	2.369	1.477 ~ 3.800	**3.469×10^{-4}**	†
Vascular invasion (Negative versus Positive)	3.339	1.884 ~ 5.916	3.621×10^{-5}	2.212	1.168 ~ 4.191	**0.015**	†	4.992	2.719 ~ 9.167	2.158×10^{-7}	3.444	1.518 ~ 7.812	**3.417×10^{-4}**	†
HVB infective state (Negative versus Positive)	1.225	0.710 ~ 2.114	0.466					1.112	0.809 ~ 1.527	0.514				
Hepatic cirrhosis (Negative versus Positive)	0.981	0.674 ~ 1.427	0.918					1.143	0.744 ~ 1.758	0.541				
TNM stage (I–II versus III–IV)	2.398	1.592 ~ 3.611	2.845×10^{-5}	1.859	1.206 ~ 2.867	**0.005**	†	2.751	1.747 ~ 4.332	1.261×10^{-5}	1.579	0.927 ~ 2.691	0.093	†
GLI2-FoxM1-KIF20A(–) versus other	2.064	1.347 ~ 3.162	0.001	2.069	1.167 ~ 3.668	**0.013**	*	1.954	1.191 ~ 3.207	0.008	1.677	1.013 ~ 2.777	**0.045**	*
GLI2 expression (low versus high)	2.257	1.545 ~ 3.298	2.597×10^{-5}	2.041	1.383 ~ 3.011	**3.237×10^{-4}**	*	2.418	1.561 ~ 3.746	7.722×10^{-5}	2.066	1.319 ~ 3.237	**0.002**	*
FoxM1 expression (low versus high)	2.114	1.433 ~ 3.119	1.611×10^{-4}	1.818	1.213 ~ 2.726	**0.004**	*	2.287	1.454 ~ 3.596	3.406×10^{-4}	1.897	1.187 ~ 3.030	**0.007**	*
KIF20A expression (low versus high)	2.290	1.554 ~ 3.374	2.793×10^{-5}	1.933	1.292 ~ 2.893	**0.001**	*	2.750	1.736 ~ 4.356	1.638×10^{-5}	2.130	1.319 ~ 3.440	**0.002**	*

ALB,albumin ;AFP,alpha-fetoprotein;CI,confidence interval;HR,hazard ratior;HBV,hepatitis B virus;TNM,tumor-node-metastasis.
†Each variable other than three protein status is adjusted with triple-negative vs others.
*Variables regarding three protein status were separately analyzed adjusting for other variables (Primary tumor size,Histologic Grade,Vascular invasion).
